# Enhancing dementia and cognitive decline detection with large language models and speech representation learning

**DOI:** 10.3389/fninf.2025.1679664

**Published:** 2025-12-05

**Authors:** Karol Chlasta, Piotr Struzik, Grzegorz M. Wójcik

**Affiliations:** 1 Department of Management in Networked and Digital Societies, Kozminski University, Warsaw, Poland; 2 Department of Artificial Intelligence, WarsawIQ, Warsaw, Poland; 3 Department of Neuroinformatics and Biomedical Engineering, Maria Curie-Sklodowska University in Lublin, Lublin, Poland

**Keywords:** dementia detection, natural language processing, large language models, transformer, machine learning, speech-based biomarkers, speech representation learning, cognitive screening

## Abstract

Dementia poses a major challenge to individuals and public health systems. Detecting cognitive decline through spontaneous speech offers a promising, non-invasive avenue for diagnosis of mild cognitive impairment (MCI) and dementia, enabling timely intervention and improved outcomes. This study describes our submission to the PROCESS Signal Processing Grand Challenge (ICASSP 2025), which tasked participants with predicting cognitive decline from speech samples. Our method combines eGeMAPS features from openSMILE, HuBERT (a self-supervised speech representation model), and GPT-4o, OpenAI's state-of-the-art large language model. These are integrated with the custom LSTM and ResMLP neural networks, and supported by Scikit-learn regressors/classifiers for both cognitive score regression and dementia classification. Our regression model based on LightGBM achieved an RMSE of 2.7775, placing us 10th out of 80 teams globally and surpassing the RoBERTa baseline by 7.5%. For the three-class classification task (Dementia/MCI/Control), our LSTM model obtained an F1-score of 0.5521, ranking 20th of 106 and marginally outperforming the best baseline. We trained models on speech data from 157 study participants, with independent evaluation performed on a separate test set of 40 individuals. We discoved that integrating large language models with self-supervised speech representations enhances the detection of cognitive decline. The proposed approach offers a scalable, data-driven method for early cognitive screening and may support emerging applications in neuropsychological informatics.

## 1 Introduction

Dementia represents a major public health challenge in developed nations, where the proportion of elderly individuals is steadily increasing. Among its causes, Alzheimer's disease (AD) is the most prevalent, accounting for 60%–80% of cases. In 2019, an estimated 57.4 million people worldwide were living with dementia—a figure projected to rise to 152.8 million by 2050 (Nichols et al., 2022).

Dementia is a leading cause of disability among those aged 60 and above, with prevalence rates in Europe rising exponentially with age—reaching 5.05% (95% CI, 4.73–5.39), and notably higher in women (7.13%) than men (3.31%) (Niu et al., 2017). According to the Global Burden of Disease Study 2021, neurological conditions, including dementia, are among the foremost contributors to disability globally (Ferrari et al., 2024).

This issue is particularly significant in European countries. For example, Poland is projected to experience a doubling in the number of people with dementia, from 525,084 in 2018 to 1,075,099 by 2050, despite an overall population decline. This increase, which slightly exceeds the European average, is driven by rapid growth in the elderly population, especially those over 80 years of age (Georges et al., 2020). Cognitive impairment encompasses deficits in memory, language, attention, and executive function that surpass normal aging, often leading to diminished independence and adverse outcomes such as increased falls, hospitalisations, financial difficulties, and heightened caregiver burden (Fowler et al., 2025).

Early detection is therefore essential, as timely diagnosis enables interventions that may slow cognitive decline and delay or prevent progression to dementia (Fowler et al., 2025; Ruzi et al., 2025). Early identification also allows clinicians to address reversible causes, optimize management of comorbidities, mitigate safety risks, and preserve patients' quality of life (Fowler et al., 2025). Automated analysis of speech has emerged as a non-invasive, cost-effective method for detecting cognitive decline, facilitating rapid and frequent monitoring without the need for specialist personnel (König et al., 2015).

Recent advances in voice-based cognitive assessment tools have demonstrated their efficacy as early screening methods. Neurodegenerative processes often manifest as subtle changes in speech and language, even at prodromal stages of dementia. Numerous studies report that speech and voice biomarkers can distinguish cognitively impaired individuals from healthy older adults with high accuracy, often around 80% for mild impairment (Martínez-Nicolás et al., 2021). Furthermore, clinical evaluations indicate high acceptance of voice-based screening among older adults (Ruzi et al., 2025). For instance, a recent mobile application for MCI achieved performance comparable to standard in-person tests, with approximately 86% user acceptance (Ruzi et al., 2025).

Speech is a well-established early indicator of cognitive deficits, including dementia (Bucks et al., 2000). Detection approaches frequently combine acoustic and linguistic feature extraction, achieving binary classification accuracy on brief spontaneous speech samples of up to 88%, with recall rates as high as 0.92 (Jarrold et al., 2014). Such methods offer the potential for fully automated, near real-time screening and can supplement traditional diagnostic processes (Weiner et al., 2016). Speech-language pathologists contribute expertise in discourse coherence, fluency, and pragmatic use—features sensitive to early-stage dementia (Boschi et al., 2017).

Dementia in speech is often detected through multi-step data processing, including voice activity detection, speaker diarisation, and extraction of acoustic and/or linguistic features. Studies have shown that speech segments as short as 2.5 min can be informative, with optimal results achieved using segments between 10 and 15 min (Weiner et al., 2018). Deep convolutional neural networks have significantly advanced the field (Krizhevsky et al., 2012; Cheplygina et al., 2019), and transfer learning consistently improves performance across a range of tasks (Kornblith et al., 2019).

Incorporating clinical insights from neuropsychologists is crucial when developing speech-based biomarkers for early dementia detection. Neuropsychologists are adept at interpreting how cognitive deficits manifest in natural language, particularly in areas such as discourse coherence, semantic content, and executive control. For example, (Mueller et al., 2018) demonstrated that individuals with subclinical cognitive impairment exhibited measurable declines in connected speech fluency and semantic richness prior to deficits appearing on conventional neuropsychological tests. Similarly, associations have been observed between amyloid-beta positivity and accelerated decline in word-level content during spontaneous speech, highlighting the value of neuropsychological expertise in contextualizing speech biomarkers (Mueller et al., 2021).

Recently, large language model (LLM)-aided feature engineering has been employed to predict AD and related dementias in an explainable and accurate manner. Kashyap et al. (2025) utilized OpenAI's GPT-4 to extract patient concept features from the Oxford Textbook of Medicine.

These findings highlight the potential of voice-based digital tools as accurate, scalable, and patient-friendly solutions for early detection and ongoing monitoring of cognitive impairment. The development and application of algorithms and data science methods within neuropsychological informatics is rapidly advancing, driven by the need for non-invasive, accessible, and ecologically valid approaches. The ADReSS (Alzheimer's Dementia Recognition through Spontaneous Speech) challenge series, held at Interspeech conferences in 2020 and 2021, exemplifies this progress, focusing on robust, generalisable models suitable for real-world clinical deployment (Luz et al., 2021b).

A growing body of research demonstrates that effective dementia detection can be achieved by combining acoustic and linguistic features extracted from spontaneous speech. Linguistic analysis, whether derived from automatic speech recognition (ASR) output or manual transcripts, has reliably identified dementia-related changes (Weiner et al., 2017). However, studies indicate that integrating acoustic and linguistic modalities yields only modest improvements over using either approach alone (Cummins et al., 2020; Rohanian et al., 2020), highlighting both the promise and current limitations of multi-modal speech analysis.

Given these challenges and opportunities, this study aims to:

- Develop and evaluate novel multimodal approaches combining speech representations with large language model-derived features for automated dementia and cognitive decline detection.
- Assess the performance of these methods using a standardized benchmark dataset that enables direct comparison with state-of-the-art approaches in speech-based dementia and cognitive declibe detection.
- Compare our proposed methods against established baselines to demonstrate their effectiveness for early cognitive screening.
- Test new LLM-derived features that can inform healthcare professionals about language and speech patterns associated with cognitive decline.

## 2 Methods

**Figure 1:**
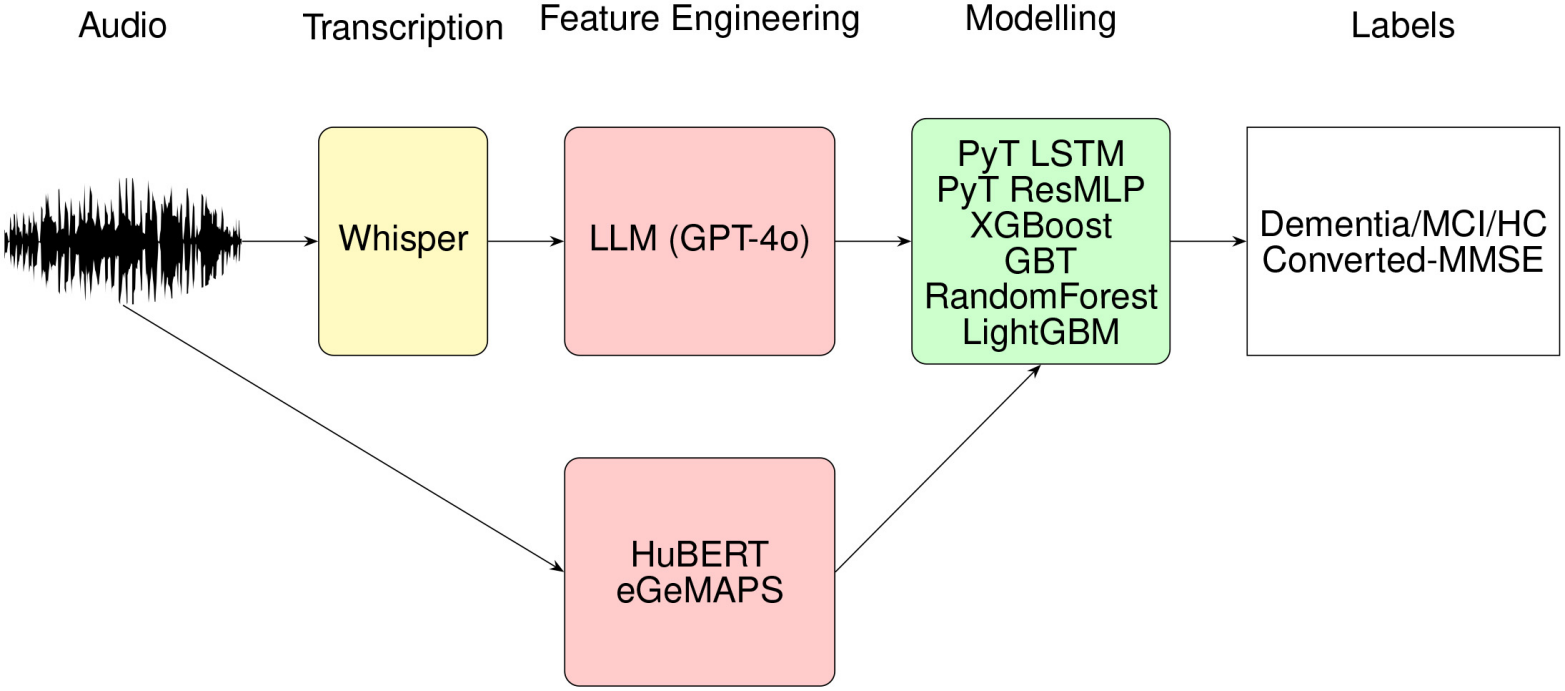
The general flow diagram for the methods proposed in this study.

In this section, we present our proposed methods for early-stage dementia detection, which leverage acoustic, paralinguistic, and pre-trained features. The overall workflow is illustrated in Figure 1 and described in detail below.

Our approach builds upon prior work by Chlasta and Wołk (2021), who introduced a two-step classification framework for detecting cognitive impairment due to AD. Their method utilized VGGish, a deep pre-trained TensorFlow model, as an audio feature extractor, followed by classical machine learning classifiers implemented in Scikit-learn. This approach achieved 59.1% accuracy—approximately 3% higher than the best-performing baseline binary classification models using acoustic features from the ADReSS challenge. Additionally, they proposed DemCNN, a convolutional neural network trained directly on raw waveforms, which reached an accuracy of 63.6%, outperforming the baseline by 7%. These findings suggest that transfer learning with pre-trained audio models (such as VGGish) can be more effective than handcrafted acoustic features in dementia detection tasks.

We also draw upon subsequent work by Chen et al. (2021), who explored multimodal approaches by integrating both acoustic and linguistic information. They extracted a wide range of acoustic features (MFCCs, GeMAPS, eGeMAPS, ComParE-2016, and IS10-Paraling) and combined them with linguistic features derived from ASR transcriptions, including LIWC and contextual embeddings from Bidirectional Encoder Representations from Transformers (BERT). Using various fusion strategies and an ensemble of the top 10 classifiers based on training performance, their model achieved a binary classification accuracy of 81.69% on the test set—exceeding the baseline of 78.87%. Importantly, their findings highlight the value of combining acoustic and textual modalities, even when ASR introduces transcription noise, and demonstrate that ensemble learning can significantly boost both accuracy and robustness in AD detection.

We participate in both tasks of 2025 ICASSP PROCESS Signal Processing Grand Challenge (Grand Challenge at ICASSP 2025, 2025). The classification task involves developing models to distinguish between three groups of patient speech [Healthy Control (HC), MCI, or Dementia groups] using the PROCESS dataset, while the regression task predicts the corresponding MMSE score of speakers from their speech.

The challenge organizers provided six baseline machine learning models utilizing both acoustic and linguistic features for the analysis of spontaneous speech pathology. Acoustic approaches included emobase (Eyben et al., 2010), ComParE 2013 (Eyben et al., 2013), Multi-resolution Cochleagram features (MRCG) (Chen et al., 2014), the Geneva minimalistic acoustic parameter set (eGeMAPS) (Eyben et al., 2015), and a minimal feature set (Luz, 2017).

Our approach is designed as a screening tool for early detection of cognitive decline, rather than for formal clinical diagnosis. We present models supporting both the classification and regression tasks, and compare our results to respective baselines in Tables 1, 2.

**Table 1:** Our approaches (best in bold) vs the best baseline accuracy classification results (on the test set) for the Cookie Theft (CT) task, semantic fluency, phonemic fluency (VF), and their combination (CT+VF) are presented in terms of accuracy (Acc.), macro-precision (Prec.), macro-recall (Rec.), and macro-F1 score, all reported as percentages.

**Model No**.	**Features**	**Prompt**	**Acc**.	**Prec**.	**Rec**.	**F1**
1-ResMLP	eGeMAPS	CT+VT	59.4	17.9	58.6	27.3
2-LSTM	**HuBERT+LLM**	CT+VT	58.7	52.6	58.7	**55.2**
3-XGBoost	HuBERT	CT	68.1	52.1	55.1	53.5
Baseline 1	SVC (eGeMAPS)	CT	57.5	53.5	61.2	55.0
		VT	45.0	38.8	39.1	38.3
		CT+VT	50.0	41.7	42.3	41.7
Baseline 2	RFC (eGeMAPS)	CT	60.0	71.7	49.9	53.3
		VF	52.5	33.1	35.9	33.9
		CT+VF	52.5	66.1	43.9	47.4
Baseline 3	RoBERTa-Classifier	CT	52.5	36.1	39.7	36.8
		VF	55.0	35.6	38.1	35.6
		CT+VF	52.5	32.2	35.9	32.9

Our approach (HuBERT+LLM) in bold vs the baselines using acoustic features (eGeMAPS) and RoBERTa.

**Table 2:** Our results on the regression task using the PROCESS test set.

**Model No**.	**Features**	**Prompt**	**RMSE**
Model 1-GBT	HuBERT+LLM	CT+VT	3.3877
Model 2-RandomForest	eGeMAPS	CT+VT	5.2870
Model 3-LightGBM	**HuBERT+LLM**	CT+VT	**2.7775**
Baseline Model 1	SVR (eGeMAPS)	CT	4.4000
Baseline Model 2	RFR (eGeMAPS)	CT+VF	3.1700
Baseline Model 3	RoBERTa-Regression	CT+VF	2.9850

Our approach (HuBERT+LLM) in bold vs the baselines using acoustic features (eGeMAPS) and RoBERTa.

In our approach, we analyse a comprehensive set of acoustic and linguistic indicators to detect cognitive decline. Acoustic features such as prosodic variations, articulation rate, speech rate, pause frequency and duration, pitch variability, articulation precision, and spectral characteristics are extracted using HuBERT. Linguistic markers—including lexical diversity (e.g., type-token ratio), syntactic complexity (e.g., mean length of utterance), semantic coherence, and discourse organization—are derived from GPT-4o-generated transcriptions. These features are known to correlate with cognitive decline and serve as potential identifiers of dementia and MCI. The measures selected are consistent with observed impairments in dementia, such as impoverished vocabulary, reduced syntactic structure, and topic drift, and align with established neuropsychological constructs for speech and language assessment.

All preprocessing steps—including feature extraction via HuBERT and transcription using Whisper—were carried out on a local workstation equipped with 32 GB RAM and a 12th Generation Intel Core i7-12700H processor (2.3 GHz, 14 cores, 20 logical threads). While GPU acceleration was assessed using an NVIDIA GeForce RTX 3060 Laptop GPU (6 GB VRAM), it did not yield significant gains in processing speed for the selected tasks. Experimental workflows followed an AutoML strategy (Dataiku, 2025), employing well-established libraries such as Scikit-learn (Pedregosa et al., 2011), XGBoost (Chen and Guestrin, 2016), and LightGBM (Ke et al., 2017), alongside custom neural network models implemented with PyTorch (Paszke et al., 2019).

We adopted the official PROCESS dataset split, assigning 80% of participants (*n* = 157; codes 001–157) to the training set and the remaining 20% (*n* = 40; codes 001–040) to the test set.

### 2.1 Dataset

We utilized the dataset provided by the PROCESS Signal Processing Grand Challenge (Tao et al., 2025), which was divided into training and test sets. The training set comprised 926 MB of data from 157 participants, each represented by a unique folder (Process-rec-XXX) containing audio recordings in .wav format and manual transcriptions from three neuropsychological tasks:

- **Semantic Fluency task:** Participants were asked, “Please name as many animals as you can in a minute.” This task is analogous to naming tasks in standard cognitive assessments, primarily evaluating language abilities and naming skills to detect potential issues in language comprehension and expression.
- **Phonemic Fluency task:** Participants were instructed, “Please say as many words beginning with the letter ‘P' as you can. Any word beginning with ‘P' except for names of people such as Peter, or countries such as Portugal.” A one-minute time limit was imposed. This task is similar to those used in cognitive assessments to test verbal fluency and executive functions related to language.
- **Cookie Theft picture task:** Participants were prompted to describe a visual scene from the picture, evaluating spontaneous narrative speech and language coherence.

The corpus provides diagnostic class labels alongside cognitive assessment scores. Several standardized measures are included, such as the Montreal Cognitive Assessment (MoCA) (Nasreddine et al., 2005), Mini-Cognitive Examination (MCE) (Vilalta-Franch et al., 1996), and Alzheimer's Cognitive Examination-III (ACE-III) (Hsieh et al., 2013), reflecting differences in clinical practice across dementia and stroke pathways. Additionally, a unified Mini-Mental State Examination (MMSE) (Cockrell and Folstein, 2002) score is provided for some participants, derived by converting MoCA, MCE, or ACE-III scores. This converted MMSE score forms the basis of the regaression task, while the diagnostic labels support the classification task.

In addition to audio and transcripts, the dataset included a metadata file (dem-info.csv) providing subject-level demographic and clinical information. This metadata comprised the dataset partition (train or development), diagnostic class (HC, MCI, or Dementia), gender, age, and cognitive status measured by the MMSE. Where MMSE scores were not directly available, they were estimated from MoCA or ACE-III assessments using validated conversion formulas (Fasnacht, 2023).

Among the 157 training participants, 82 were labeled HC, 59 MCI, and 16 Dementia. MMSE scores were available for 69 individuals. Age information was missing for 24 participants and was imputed using the average age across the dataset (66 years), marked with an asterisk. For the remaining 133 individuals, the age range was 23–94 years. Table 3 presents the demographic summary for the PROCESS training and development dataset. This dataset was used exclusively for training our models.

**Table 3:** Demographic summary for the PROCESS training and development dataset.

**PROCESS dataset statistics**	**Value**
Dementia cases	16
MCI cases	59
Healthy controls	82
Total participants	157
Female participants	81
Male participants	75
Participants with age info	133
Min Age	23
Max Age	94
MMSE scores available	69
Mean MMSE	27.36

A separate test dataset, comprising 236 MB of data from 40 additional study participants, was provided for evaluation. The test dataset contains audio recordings for the same three neuropsychological tasks, but does not include manual transcriptions. This design ensures that model performance is assessed on previously unseen data, reflecting real-world deployment scenarios. Table 4 presents the demographic summary for the PROCESS test dataset.

**Table 4:** Demographic summary for the PROCESS test dataset.

**PROCESS dataset statistics**	**Value**
Total participants	40
Female participants	21
Male participants	19
Participants with age info	40
Min age	45
Max age	86

For each participant in the test set, our classification models produced a diagnostic label (Healthy Control, Mild Cognitive Impairment, or Dementia), wheras our regression models predicted the MMSE score for each individual.

#### 2.1.1 Acoustic features (eGeMAPS)

To extract acoustic biomarkers relevant to cognitive state, we employed the openSMILE toolkit (Eyben et al., 2010) configured with the extended Geneva Minimalistic Acoustic Parameter Set (eGeMAPSv02), a standardized set of 88 features designed for paralinguistic and clinical voice analysis (Eyben et al., 2015).

Our pipeline processed three distinct audio recordings per subject—CT, PF, and SF—each located within structured directories corresponding to a different speech task. Using openSMILE, we computed summary statistics (functionals) over low-level descriptors such as fundamental frequency (F0), shimmer, jitter, harmonic-to-noise ratio (HNR), spectral flux, and Mel-frequency cepstral coefficients (MFCCs), all of which have demonstrated high discriminative power in dementia-related speech studies (König et al., 2015; Martínez-Nicolás et al., 2021).

In parallel, we used Parselmouth (Jadoul et al., 2018), a Python interface to Praat, to extract additional temporal and perturbation-based features—mean pitch, pitch variation, mean intensity, local jitter, local shimmer, and mean HNR—which supplement the eGeMAPS set with clinically interpretable measures of vocal fold stability and voice quality.

All extracted features were tabulated for downstream analysis. This dual-extraction strategy is consistent with current best practices in computational paralinguistics and neurocognitive speech analysis, which emphasize the integration of spectral, prosodic, and phonatory metrics (Schuller et al., 2013; Eyben et al., 2015).

#### 2.1.2 HuBERT

We utilized HuBERT (Hsu et al., 2021), a self-supervised learning model for speech representation that has demonstrated superior performance in tasks such as automatic speech recognition and speaker verification. The pre-trained HuBERT model^1^ was used to extract high-level features from the audio recordings.

For each recording, we extracted 1,024-dimensional feature vectors using the HuBERT model. These features served as inputs to our classification and regression models. To access HuBERT, we utilized the Hugging Face Transformers library (Wolf et al., 2020), which provides a user-friendly interface for working with pre-trained models. Feature extraction was performed by passing the audio recordings through HuBERT and obtaining the representations from the final hidden layer. Specifically, HuBERT-based acoustic embeddings were extracted from the recordings of all three tasks (SF, PF, and CT) and concatenated to form a unified feature representation for each individual. These representations were subsequently used in the modeling tasks.

#### 2.1.3 LLM (Whisper + GPT-4o)

As part of our analysis, we explicitly extracted and scored clinically relevant speech and language features from each task using large language models (LLMs). For every participant, LLMs were employed to evaluate the following dimensions:

- **Cookie theft description (CT):** Content accuracy, language fluency, grammar and syntax, organization, and awareness of key details.
- **Phonemic fluency task (PF):** Phonemic fluency, repetition errors, intrusion errors, pace and effort, and total valid words.
- **Semantic fluency task (SF):** Semantic fluency, repetition errors, intrusion errors, pace and effort, semantic clustering and switching, and total valid items.

These dimensions were selected based on neuropsychological literature and clinical practice, reflecting core aspects of cognitive and linguistic function affected by dementia and MCI. Each feature was scored on a scale from 0 (full mental capabilities) to 10 (heavy dementia) by prompting the LLM with standardized templates (see Table 5), ensuring that our feature set is both interpretable and clinically meaningful. Specifically, for the Cookie Theft Description (CT) task, we extracted content accuracy, language fluency, grammar and syntax, organization, and awareness of key details; for the Phonemic Fluency Task (PF), we extracted phonemic fluency, repetition errors, intrusion errors, pace and effort, and total valid words; and for the Semantic Fluency Task (SF), we extracted semantic fluency, repetition errors, intrusion errors, pace and effort, semantic clustering and switching, and total valid items. All these features were rated on a scale from 0 (worst score) to 10 (best score) and subsequently used as input to our classification and regression models, with extraction guided by the LLM prompts presented in Table 5.

**Table 5:** LLM prompt templates for extracting features from three speech tasks: Cookie Theft (CT), Phonemic Fluency (PF), and Semantic Fluency (SF).

**Task**	**Prompt template**
CT (Cookie theft description)	Evaluate the cognitive capabilities of the person based on The Cookie Theft description task from Mini-Mental State Examination (MMSE). Provide a score for key aspects like *Content Accuracy, Language Fluency, Grammar and Syntax, Organization, Awareness of Key Details* and a summary score from 0 (full mental capabilities) to 10 (heavy dementia), along with a brief reasoning for the score. Here is the transcription with patient's response marked as Pat: and other persons' responses that should be dismissed marked as Oth:
PF (Phonemic fluency test)	Evaluate the cognitive capabilities of the person based on Phonemic Fluency Test task from Mini-Mental State Examination (MMSE). Provide a score for key aspects like *Phonemic Fluency, Repetition Errors, Intrusion Errors, Pace and Effort, Total Valid Words* and a summary score from 0 (full mental capabilities) to 10 (heavy dementia), along with a brief reasoning for the score. Here is the transcription with patient's response marked as Pat: and other persons' responses that should be dismissed marked as Oth:.
SF (Semantic fluency test)	Evaluate the cognitive capabilities of the person based on Semantic Fluency Test task from Mini-Mental State Examination (MMSE). Provide a score for key aspects like *Semantic Fluency, Repetition Errors, Intrusion Errors, Pace and Effort, Semantic Clustering and Switching, Total Valid Items* and a summary score from 0 (full mental capabilities) to 10 (heavy dementia), along with a brief reasoning for the score. Here is the transcription with patient's response marked as Pat: and other persons' responses that should be dismissed marked as Oth:.

None of these tasks are part of the MMSE. Prompts were designed to assess cognitive-linguistic performance with scores from 0 (no impairment) to 10 (severe impairment).

For feature extraction using a LLM, we first transcribed all audio recordings from the PROCESS study participants using Whisper (Radford et al., 2022), a state-of-the-art ASR system developed by OpenAI. Whisper is a transformer-based model trained on a large, diverse corpus of multilingual audio data, enabling accurate transcription across various languages and dialects. Transcriptions were generated using the official Whisper library,^2^ and subsequently served as input to OpenAI's LLM (Hurst et al., 2024) for downstream analytical tasks.

### 2.2 Classification method

We conducted a two-step experimental procedure to detect dementia and MCI, as illustrated in Figure 1. For the classification task, we developed models that leveraged both acoustic and language-derived features to predict cognitive impairment. These were then combined with 12 interpretable, high-level features derived from large language models (LLMs), encompassing measures such as CT Language Fluency, PF Total Valid Words, and SF Repetition Errors.

Classification models were trained using several algorithms, including Support Vector Machines (SVM) with a radial basis function (RBF) kernel, Gradient Boosted Trees (GBT), LightGBM, and XGBoost, implemented via Dataiku's DSS platform (Dataiku, 2025). All classifiers utilized the full feature set, which comprised HuBERT-based embeddings from verbal fluency tasks (SF, PF) and a discourse elicitation task (CT), along with 12 high-level features derived from LLM. Performance was assessed on held-out test data to ensure generalizability. To enhance robustness, ensemble learning techniques were applied by aggregating predictions from classifiers trained on individual feature subsets (e.g., HuBERT_Features_SF, PF, and CT), yielding a total of nine model variants. Final decisions were derived through decision-level fusion. In cases of prediction ties, the label “dementia” was assigned to mitigate the risk of under-diagnosis—consistent with clinical practice, where false positives (Type I errors) are generally considered less harmful than false negatives (Type II errors).

We further implemented a neural network-based classification model using the LSTMSpeechClassifier, a custom PyTorch module designed to handle high-dimensional speech features. The architecture begins with a batch normalization layer to standardize input features, followed by a two-layer Long Short-Term Memory (LSTM) network with 256 hidden units, which captures temporal dependencies and sequential dynamics inherent in the speech data. The output sequence from the LSTM is passed through a fully connected linear layer to generate logits for three diagnostic categories (output_dim = 3).

During the forward pass, input features are normalized and reshaped to incorporate a temporal sequence dimension before being processed by the LSTM; the final hidden state is then mapped to class predictions via the classifier layer. This design enables the model to combine robust feature-level normalization with sequential modeling of cognitive status. The training process converged within 20 epochs, and early stopping based on validation loss was employed to mitigate overfitting.

We also implemented a custom neural network model termed ResidualMLP, developed as a PyTorch module for processing high-dimensional speech features using a deep multilayer perceptron (MLP) architecture enhanced with residual connections. The network comprises sequential fully connected layers with decreasing hidden dimensions of 512, 384, 256, and 128 units. Each layer is followed by batch normalization and non-linear activation to ensure stable learning and effective feature transformation. Residual connections are introduced between layers of matching dimensions to facilitate gradient flow and mitigate vanishing gradient issues, thereby improving training dynamics and model convergence. This architecture enables efficient learning from complex speech embeddings by leveraging deep nonlinear transformations while retaining stability through architectural design.

### 2.3 Regression method

For the regression task, the goal was to predict participants' cognitive performance, quantified via the Converted-MMSE score, based solely on speech-derived features. The input representation combined low-level acoustic embeddings and high-level linguistic attributes. Specifically, HuBERT-based embeddings were extracted from three speech tasks: a discourse elicitation task (CT) and two verbal fluency tasks (SF, PF). Each task produced a 1024-dimensional feature vector. In parallel, twelve cognitively and linguistically informative features derived from large language models (LLMs) were incorporated, including syntactic complexity from CT (e.g., grammar and clause structure), semantic fluency metrics from SF, and repetition patterns from PF. All features were concatenated into a single high-dimensional input vector, enabling the model to jointly leverage fine-grained acoustic signals and abstract semantic-linguistic cues for cognitive status estimation.

To assess performance across various algorithmic families, we employed an AutoML strategy (Dataiku, 2025), which facilitated systematic hyperparameter tuning and model selection. The following regression model families were evaluated in this study: Gradient Boosting Trees (GBT), Random Forests, and LightGBM.

All regression models were evaluated on held-out test data to ensure generalizability. Hyperparameter optimization for tree-based methods was performed via cross-validation, with a focus on minimizing Root Mean Squared Error, while minimizing overfitting. Since the official Challenge test labels were withheld, we conducted post-hoc analyses on the released validation subset (40 samples, of which 21 had Converted-MMSE scores) to further compare models and assess demographic effects.

Convergence behavior for the tree-based models—namely LightGBM and XGBoost—was managed internally via early stopping mechanisms provided by the Dataiku Data Science Studio platform (Dataiku, 2025). As these models are based on decision tree ensembles, they do not rely on epoch-based training. Instead, hyperparameter tuning was conducted using cross-validation, focusing on optimizing performance metrics while mitigating overfitting. LightGBM and XGBoost dynamically determine the optimal number of boosting iterations based on validation set performance, thus eliminating the need for manual control over training duration and ensuring efficient convergence.

We employed three complementary feature streams. First, HuBERT embeddings (Base model, 12 transformer layers) were extracted from speech recordings, capturing contextualized acoustic–phonetic information. Second, LLM-derived textual embeddings were obtained by prompting transcripts with both cognitive task (CT) and verbal task (VT) contexts; embeddings were generated using a transformer-based encoder and concatenated across prompts. Third, eGeMAPS acoustic features (88-dimensional, hand-crafted low-level descriptors) were extracted using the openSMILE toolkit. For composite systems, these representations were concatenated without additional dimensionality reduction, allowing the downstream models to determine feature relevance.

For modeling, we used three classical machine learning approaches with fixed configurations: (1) Gradient Boosted Trees (GBT) with 300 estimators, maximum depth of 6, and learning rate 0.1; (2) Random Forest (RF) with 200 estimators and maximum depth of 12; and (3) LightGBM with 500 boosting rounds, maximum depth of 7, and learning rate 0.05. All models used default class weighting and early stopping where available. Transformer-based features were used as frozen embeddings (no fine-tuning). Detailed hyperparameters are provided in Supplementary Table 11.

## 3 Experiments and results

Table 1 presents a summary of our classification results on the multi-task speech data from the PROCESS test set, where participants were categorized into HC, MCI, or Dementia groups. In addition to the F1 score, we report standard metrics including accuracy, precision, and recall. Our regression results—predicting participants' cognitive performance as measured by the MMSE score (Cockrell and Folstein, 2002)—are shown in Table 2.

Overall, the findings highlight the efficacy of multi-modal feature integration, specifically combining HuBERT embeddings with LLM-derived linguistic features (HuBERT+LLM) in improving models performance in relation to baselines, across both classification and regression tasks.

HuBERT and LLM features (transformer-based) played a central role in capturing higher-order temporal and semantic structure, which proved critical for forecasting cognitive scores, whereas eGeMAPS served as a traditional baseline.

In regression analysis (Table 2), LightGBM trained on HuBERT+LLM features yielded the lowest RMSE (2.7775), demonstrating superior prediction accuracy relative to models relying solely on handcrafted acoustic features (e.g., SVR RMSE = 4.4000) or RoBERTa-based regression models (RMSE = 2.9850).

In the regression task, our best LightGBM Model 3 trained on HuBERT embeddings combined with LLM-derived features—achieved a Root Mean Squared Error (RMSE) of 2.7775, ranking 10th out of 80 submissions. This represents a 7.5% improvement over the best baseline model, which was based on RoBERTa and reported an RMSE of 2.9850.

SHAP-based feature importance analysis of our LightGBM model (Supplementary Figure 2) shows that individual features exert only modest influence, with the most informative (e.g., SFT Semantic Clustering and Switching, PFT Total Valid Words, CTD Awareness of Key Details) contributing 2%–3% each. A larger number of semantic fluency and error-related variables (e.g., intrusion errors, phonemic fluency) contributed at the 1%–2% level. Their presence among the top-ranked predictors suggests that even relatively low-weighted LLM- and HuBERT-derived signals provide complementary value when integrated into the multimodal framework.

In the classification setting (Table 1), the LSTM-based model utilizing HuBERT+LLM features achieved the highest macro-F1 score (55.2%) and recall (58.7%), outperforming conventional baselines such as RoBERTa-Classifier (F1 = 32.9%) and Support Vector Classifier (SVC; F1 = 41.7%). Although ResidualMLP and XGBoost configurations demonstrated competitive results, they fell short of the LSTM in terms of precision and recall.

In the three-class classification task distinguishing between Dementia, MCI, and HC groups our best LSTM Model 2, trained on HuBERT embeddings combined with LLM-derived features, achieved an F1-score of 0.5521. This performance ranked 20th out of 106 submissions in the PROCESS Challenge, narrowly exceeding the best baseline model (SVC using eGeMAPS features) by 0.4%.

The feature importance analysis revealed that HuBERT embeddings overwhelmingly influenced the model's decision-making process, contributing 75.7% of the total predictive power, and demonstrated robust discriminative capacity for detecting cognitive decline. Principal component analysis (PCA) identified clear clustering patterns, with the first 20 components explaining 82% of the variance. Specific dimensions (42, 156, 203, 387, 512) emerged as particularly salient. Unlike interpretable acoustic features, these learned representations encoded abstract speech characteristics—such as phonetic content, prosodic dynamics, and temporal structure—within a distributed latent space. The t-SNE visualization confirmed non-linear separability between cognitive states, suggesting that self-supervised speech models can autonomously uncover clinically relevant biomarkers. This capability provides a valuable complement to traditional acoustic analysis in the context of dementia detection. More detais on the PCA of HuBERT-derived speech embeddings can be found in Supplementary Figure 3.

LLM-derived linguistic features contributed 13.5% to the model's predictive capacity, with GrammarSyntax (5.41%) and ContentAccuracy (2.70%) showing positive importance values, suggesting that preserved grammatical structure and content accuracy are protective factors against dementia classification. Conversely, AwarenessOfKeyDetails (–2.70%) showed negative importance, indicating that reduced awareness contributes to dementia detection. The remaining 10.8% of predictive power came from demographic and cognitive assessment features, most notably Converted-MMSE (–16.22%), which showed the strongest individual contribution across all feature categories. This multimodal approach demonstrates that while HuBERT speech representation embeddings provide the primary discriminative information, the integration of linguistic content analysis and established cognitive assessments creates a comprehensive framework for automated dementia detection. More details on LLM feature importance scores for our best LSTM-based classification model 2 can be found in Supplementary Figure 4.

Post-hoc validation analyses (21 regression cases, 40 classification cases) reproduced the same relative ranking as the official Challenge results: Model 3 (LightGBM, HuBERT+LLM) performed best (RMSE = 2.61 vs. 2.78 in the official test set), followed by Model 1 (GBT, HuBERT+LLM; RMSE = 1.66 vs. 3.39), with Model 2 (Random Forest, eGeMAPS) worst (RMSE = 4.57 vs. 5.29). Classification performance was modest (max accuracy 0.50, macro-F1 0.27). Models agreed on only 25% of cases, and regression predictions were weakly correlated, suggesting complementary information. The outcome of this post-hoc validation for the regression task is presented in Table 6, and for the classification task in Table 7.

**Table 6:** Comparison of regression performance (RMSE) between the official PROCESS Challenge test set and the released validation subset used for *post-hoc* analyses.

**Model**	**Official test RMSE**	***Post-hoc* validation RMSE**
Model 1 (GBT, HuBERT+LLM)	3.39	1.66
Model 2 (Random forest, eGeMAPS)	5.29	4.57
Model 3 (LightGBM, HuBERT+LLM)	2.78	2.61

Although absolute values differ due to dataset size (21 regression cases vs. the full hidden test set), the relative ranking of models is consistent.

**Table 7:** Classification performance of submitted models on the PROCESS validation test set.

**Model**	**Accuracy**	**Macro-F1**
Model 1	0.300	0.214
Model 2	0.400	0.286
Model 3	0.500	0.265

Additionally, due to mean age imputation we report stratified regression results (RMSE, MAE, *R*^2^) by gender and age group in Supplementary Table 9, along with agreement and correlation analyses between the submitted models in the Supplementary Table 10. Results indicated that predictions were more consistent for male participants (lower variance in RMSE and MAE), while female participants showed stronger correlations with ground-truth MMSE (higher *R*^2^).

Age related differences in forecasting performance were also apparent in our *post-hoc* analyses (Supplementary Table 9). Model 1 (GBT, HuBERT+LLM) achieved substantially higher explained variance for participants over 70 years (*R*^2^ = 0.60) compared to those 70 and younger (*R*^2^ < 0), whereas Model 3 (LightGBM, HuBERT+LLM) performed better in the younger group but degraded markedly for older participants (RMSE 1.60 vs. 3.91). Model 2 (Random Forest, eGeMAPS) was consistently weak, with extreme instability in the ≤ 70 group. These differences suggest that forecasting accuracy may be confounded by age, although the small sample size in the ≥70 group (*n* = 7) precludes strong statistical claims.

We also present per-class classification metrics for submitted models in Table 8 and confusion matrices for the models in Supplementary Figure 5.

**Table 8:** Per-class classification metrics for submitted models.

**Model**	**Class**	**Precision**	**Recall**	**F1**
Model 1	Dementia	0.11	0.40	0.17
	HC	0.00	0.00	0.00
	MCI	0.53	0.43	0.48
Model 2	Dementia	0.00	0.00	0.00
	HC	0.26	0.42	0.32
	MCI	0.61	0.48	0.54
Model 3	Dementia	0.00	0.00	0.00
	HC	0.20	0.08	0.12
	MCI	0.58	0.83	0.68

Models relying exclusively on acoustic features (eGeMAPS) delivered disappointing results. ResidualMLP Model 1, trained on these features, produced a mean cross-validated accuracy of 59.38% across 10 folds, with an average recall of 58.57%, precision of 17.9%, and an F1 score of 27.3%. The training loss averaged 0.2116 across folds, with fold-wise losses ranging from 0.15 to 0.37, indicating a moderately good fit and no signs of severe overfitting.

Final evaluation results for our custom audio-based classification and regression models were submitted to the 2025 IEEE International Conference on Acoustics, Speech, and Signal Processing PROCESS Grand Challenge following model retraining on the full PROCESS training set and prediction on the full test set. Classification outcomes are summarized in Table 1, while regression performance metrics are presented in Table 2. The official rankings and final performance scores were computed and verified by the PROCESS Signal Processing Grang Challenge committee.^3^

We recommend applying our two winning approaches to the neuropsychology community. For the three-class classification task (differenciating between dementia, mild ognitive impairment, and control groups), we advocate the use of our LSTM model combining HuBERT speech representation and LLM-derived features, which achieved 58.7% accuracy, 52.6% precision, 58.7% recall, and an F1-score of 55.2, outperforming the baseline. For the regression task, our LightGBM model, utilizing the same self-supervised HuBERT and LLM features, attained a Root Mean Squared Error (RMSE) of 2.7775, ranking in the top 10 globally and improving over the best baseline by 7.5%. These findings highlight the value of integrating deep acoustic and interpretable, clinically meaningful language-based features for more effective, speech-only cognitive assessment.

## 4 Discussion

The classification accuracy achieved in this dementia detection task is only slightly better than prior ADReSS challenges and exceeds the best PROCESS baseline by 0.4%. These benchmarks continue to highlight the inherent complexity of speech-based dementia classification, where improvements remain modest. Unlike earlier binary classification tasks, the PROCESS challenge introduces a three-way classification between HC, individuals with MCI, and those with general dementia, a notably more difficult task. MCI, being a heterogeneous and transitional state, presents overlapping linguistic and cognitive features with both healthy aging and early-stage dementia. Differentiating these subtle gradations demands models attuned to nuanced speech and cognition patterns—challenges that even clinical assessments struggle with. Progress in this domain holds technical significance and promises meaningful impact for clinical screening and early intervention.

Similar to our approach, (Zafar et al., 2025) utilized acoustic features for the PROCESS Challenge, specifically employing the ComParE feature set originally developed for emotion recognition (Weninger et al., 2013). These features were extracted using the openSMILE Python toolkit (Eyben et al., 2013), resulting in a high-dimensional (6,373, 1) feature vector comprising 6,373 statistical functionals derived from low-level descriptors over the full duration of each sample. In contrast, our model used a more compact set of 264 features based on the eGeMAPS configuration. Despite this difference in feature dimensionality, both approaches achieved comparable classification performance: Zafar reported 58% accuracy, while our model reached 59.4%. These results are consistent with Baseline 1 (57.5%) and Baseline 2 (60%) listed in Table 1, all evaluated on the same test set.

Notably, models trained exclusively on eGeMAPS acoustic features reported higher error rates and suboptimal classification performance. These findings reinforce that leveraging deep speech representations in conjunction with semantically enriched language features is instrumental for accurate diagnosis and cognitive severity estimation in dementia. The results advocate for the deployment of advanced architectures such as LSTM networks and gradient boosting frameworks to tackle complex neuropsychological assessment challenges effectively. The results also highlight the competitive advantage of integrating deep acoustic and semantic-linguistic representations for neuropsychological screening.

While the relatively high classification recall indicates moderate sensitivity to dementia detection, the low precision suggests a substantial false positive rate. This trend toward over-predicting dementia may be clinically acceptable in scenarios where early detection is prioritized over specificity.

Although the current system targets individuals already exhibiting signs of impairment, future work may explore its utility in identifying at-risk individuals prior to symptom onset. The dataset encompasses a broad spectrum of diagnoses, including MCI and control groups comprising healthy volunteers and individuals with non-neurodegenerative memory complaints, thereby reflecting real clinical populations.

While early detection of dementia remains clinically valuable by enabling timely interventions, lifestyle adjustments, and care planning, our study does not provide evidence for identifying pre-clinical symptoms. The model was trained on a cross-sectional dataset comprising individuals with diagnosed dementia, MCI (59 out of 157), and no impairment (24 out of 157). As such, it demonstrates potential as a scalable and accessible screening tool for observable cognitive decline, but its applicability to asymptomatic individuals or pre-clinical stages cannot be inferred without longitudinal validation.

The field of dementia detection continues to face significant limitations, including insufficient standardization, limited cross-study comparability, and a disconnect between research objectives and clinical practice. This article specifically discusses the difficulty of detecting AD using speech and language processing, particularly through AI methods. Our two proposed approaches aim to help bridge these gaps by directly addressing several of these persistent challenges.

Dementia detection faces significant challenges. As noted by de la Fuente Garcia et al. (2020), detecting AD through speech and language processing, especially with AI-based methods remains difficult. Although promising results exist, few approaches have been applied in clinical research or practice. Key limitations mentioned by the authors include poor standardization, limited comparability of findings, and a gap between research objectives and clinical relevance. Our study addresses these issues by introducing new methods, evaluating them on a benchmark dataset for comparison, and presenting AI- and LLM-based approaches to the community of clinical practioners.

Limited data availability has historically constrained research into speech-based dementia detection. To address this, the field increasingly employs transfer learning (Yosinski et al., 2014), leveraging pre-trained models as audio feature extractors for rapid classifier development, while also improving corpora focused on early-stage detection. Building on the work of (Luz et al. 2021b, a, 2024), our training dataset expands diagnostic coverage to include MCI—aligned with real-world clinical labels—and incorporates control groups with healthy individuals.

Findings from the ADReSS Challenges confirmed that linguistic systems consistently outperform acoustic ones in Alzheimer's detection (Yuan et al., 2020; Cummins et al., 2020). This is perhaps unsurprising, as the transcripts used were manually generated by human annotators and thus contained significantly less noise than the original audio recordings. However, such manual processing limits scalability, making these approaches impractical for routine clinical use.

The current PROCESS corpus includes diagnostic labels derived from several cognitive assessments commonly used in neuropsychology, such as the MoCA (Nasreddine et al., 2005), MCE (Vilalta-Franch et al., 1996), and ACE-III (Hsieh et al., 2013), alongside unified MMSE scores (Cockrell and Folstein, 2002) employed for prediction tasks. However, due to gaps in assessment coverage, only MMSE scores were consistently available for model training. Future work will explore more robust strategies for addressing missing cognitive assessment data.

Furthermore, age was available for most participants, but missing for 24 cases. For these, the PROCESS Challenge organizers applied mean imputation (66 years) to preserve sample size and maintain consistency across models. While this avoids data loss, mean imputation attenuates variance and may obscure age-related effects. Indeed, when stratifying by age at 70 years (Supplementary Table 9), we observed clear differences in regression performance: Model 1 and Model 3 achieved stable errors in the >70 group (RMSE = 1.81 and 3.91, respectively), while Model 2 was particularly unstable. In contrast, the below 70 group showed higher variance and even negative *R*^2^ values, suggesting that imputing many samples at exactly 66 years may have blurred genuine age effects. Notably, only 2 participants in the imputed group had MMSE scores available, further limiting interpretability. These observations highlight the importance of richer demographic coverage and complete metadata in future datasets, to better disentangle potential confounding effects of age and sex on model predictions.

Additionally, including young adults in the control group may bias the classification model, as age-related acoustic differences can overshadow disease-specific vocal changes. The model may thus learn to distinguish age rather than detect dementia and cognitive decline.

Another limitation relates to automatic speech recognition. We used Whisper to generate transcripts, but did not calculate word error rates (WER) due to time constraints during the Challenge. In practice, we observed systematic error modes: Whisper occasionally appended random hallucinated words at the end of transcripts (which we corrected manually) and sometimes inserted invented content in place of extended silences (more difficult to detect). While the most obvious hallucinations were removed prior to scoring, a comprehensive WER analysis was not conducted. Future work should quantify transcription errors per task and assess their downstream impact on LLM-derived rubric scores and overall model performance.

We recognize the limitations in LLM-based scoring, including alignment gaps with human graders and reliance on shortcuts rather than deeper reasoning (Wu et al., 2025). Prior studies show that while LLMs adapt quickly to scoring tasks, they often resort to heuristics, bypassing the logical reasoning expected in human grading. To mitigate this, we have done a sanity check of generated grades, and used LLM-derived features only as partial inputs within our broader feature engineering framework (see Supplementary Figure 2).

Although our study is primarily technical, its findings align closely with clinically validated speech-based indicators of cognitive impairment. Future research will benefit from interdisciplinary collaboration with clinicians to deepen interpretation of both linguistic and acoustic patterns within the framework of clinical dementia diagnosis.

We hypothesized that combining modalities would improve performance, but we were only able to evaluate the full concatenated system. Ablation experiments isolating HuBERT-only, LLM-only, and eGeMAPS-only features remain future work. Our results suggest that combining representation learning features (HuBERT, LLM) with eGeMAPS may capture complementary information. However, because ablations could not be run on the hidden test set, and post-hoc analyses are limited to a smaller released subset, this conclusion should be interpreted cautiously. Nevertheless, the consistency of model ranking across both official test and validation subsets supports our interpretation. We note that our baseline models were kept close to default settings, in line with Challenge rules, and should therefore be interpreted as lower-bound references rather than fully optimized classifiers.

Our current approach utilizes LLMs to extract features from the available tasks. However, the dataset's structure constrains the full potential of LLM-driven analysis. Specifically, only the Cookie Theft task offers open-ended narrative responses suitable for rich linguistic and semantic evaluation. In contrast, the Semantic and Phonemic Fluency tasks involve listing isolated words—such as animals or words beginning with a given letter—which lack syntactic complexity and do not elicit connected speech.

To fully leverage the potential of LLMs in dementia detection, future datasets should incorporate a broader range of open-ended prompts that elicit longer, more complex responses. Such data would enable LLMs to examine advanced language features—including coherence, narrative structure, and pragmatic usage—which are often disrupted in cognitive impairment but remain inaccessible through constrained word-listing tasks. Enhancing datasets with these richer linguistic samples would improve both the sensitivity and clinical relevance of LLM-based speech analysis in dementia screening.

As model complexity and parameter count continue to increase, future research will require more extensive datasets to fully exploit the capabilities of advanced architectures. One promising direction involves assembling a large dataset of spontaneous speech samples encompassing multiple pathologies—beginning with depression and dementia in older adults. Such data would support the development of robust models capable of differentiating among various types of pathological speech. This direction is particularly justified by the documented association between late-life depression and the onset of dementia in individuals over 50 years old, supporting the hypothesis that depression may not only predict but also contribute causally to subsequent cognitive decline (Buntinx et al., 1996).

Dementia is a heterogeneous condition, with clinical profiles that vary significantly across subtypes—such as Alzheimer's disease, frontotemporal dementia—and across stages of progression. Although our current system does not explicitly differentiate between these subtypes, its use of continuous speech and language modeling enables sensitivity to the nuanced spectral patterns associated with different forms of cognitive decline. Future work may explore stratified modeling by clinical subtype or disease stage to improve diagnostic specificity and better reflect the diversity of dementia presentations.

We anticipate that the 2025 PROCESS corpus will be expanded with additional data, which—when supplemented by the existing DementiaBank's Pitt dataset (Jost and Grossberg, 1995) would yield a sufficiently large and diverse benchmark for experimenting with both custom and off-the-shelf deep neural network architectures. However, annotation quality and consistency remain key challenges that may constrain progress in automated dementia detection. Addressing these limitations will be crucial as we aim to retrain and optimize our models on larger, more representative corpora.

Looking ahead, we anticipate that the advantages of LLM-based analysis will become more evident as future datasets incorporate spontaneous speech, conversational exchanges, and longitudinal narrative samples. In our results, HuBERT embeddings provided useful acoustic representations but are optimized primarily for phonetic and prosodic patterns rather than higher-order linguistic or pragmatic features. By contrast, LLM-derived features are specifically designed to integrate semantic and discourse-level cues across extended stretches of language, enabling detection of subtle disruptions in coherence, sentence structure, and communicative intent—markers highly relevant for early dementia detection. While our current winning framework combines deep speech representation and LLM-derived features, we expect the relative contribution of LLM-based analysis to increase as datasets evolve to better reflect the complexity of naturalistic communication.

Ultimately, integrating LLM-driven speech analysis into routine health assessments presents a scalable and promising avenue for the early detection of cognitive decline in aging populations. However, successful clinical deployment will depend on rigorous validation, improved interpretability of model outputs, and close collaboration with healthcare professionals to ensure clinical relevance and trust.

This study represents a foundational effort to align machine learning-based speech analysis with core principles of neuropsychological theory. Advancing this work will require richer clinical annotations and sustained collaboration with neuropsychologists to ensure that computational metrics correspond meaningfully to established cognitive constructs and diagnostic frameworks.

## 5 Conclusion

This study introduced a multimodal framework that integrates large language model–based linguistic features with self-supervised speech representations to enhance dementia and cognitive decline detection. The proposed approach exceeded the benchmark results from the 2025 IEEE ICASSP PROCESS Grand Challenge, ranking 10th worldwide in regression and 20th in classification. Our results suggest that LLM-derived representations can complement traditional neuropsychological tools, offering new avenues for early detection, remote monitoring, and resource-efficient automated screening in clinical and research settings.

Early detection of dementia is particularly valuable, as it enables timely clinical interventions, lifestyle modifications, and planning that may delay disease progression or improve quality of life. This is supported by the inclusion in our training dataset of patients diagnosed with dementia, as well as a significant proportion of individuals with mild (59 out of 157) or no cognitive impairments (24 out of 157).

Although large language models such as GPT-4o are often considered black boxes, our analysis of linguistic outputs identifies markers associated with cognitive decline, including reduced lexical diversity, grammatical errors, and topic drift. These markers can inform clinicians about language degradation patterns without the need for manual transcription or annotation. To facilitate practical use, the system can be integrated into telehealth platforms or deployed in primary care settings for initial screening.

Importantly, this method relies solely on patient audio recordings, making it a non-invasive, low-cost, and scalable approach—ideally suited for broad deployment in clinical settings, telemedicine, or even home-based screening tools. Such accessibility could greatly enhance early dementia risk assessment, particularly in under-resourced or aging populations.

We believe this work, alongside others presented at the PROCESS Challenge, holds significant implications for neuropsychology, especially in the context of ongoing debates regarding the role of technology in assessment. As neuropsychology seeks to integrate modern tools—such as machine learning, speech analysis, and large language models—into clinical and research paradigms, our findings provide a concrete example of how technology can enhance, rather than supplant, core neuropsychological principles. Rather than simply digitizing conventional assessments or repackaging legacy tests into virtual formats, our approach demonstrates that fine-grained acoustic and linguistic features, automatically extracted from naturalistic speech, can reveal cognitive markers relevant to early dementia detection. This represents a methodological shift, moving beyond traditional test batteries toward models grounded in spontaneous behavior and continuous variables, analyzed through rigorous computational techniques.

By quantifying clinically relevant linguistic and acoustic markers of cognitive decline, this study contributes to the broader field of neuroinformatics and its application to neuropsychological assessment. Specifically, we:

- Developed a multi-modal speech-based framework integrating HuBERT and LLM (GPT-4o) for dementia and cognitive impairment detection, achieving performance surpassing strong baselines in both classification and regression tasks.
- Demonstrated that combining deep acoustic embeddings with LLM-derived linguistic features significantly enhances detection of cognitive impairment from spontaneous speech.
- Achieved a top-10 ranking in the global PROCESS Challenge regression task with a 7.5% improvement over the best baseline RMSE, and ranked 20th out of 106 in the classification task with a marginally better F1-score than existing methods.
- Showed the clinical relevance and scalability of non-invasive, speech-only models for early dementia screening, applicable across varying speech tasks and cognitive conditions.

## Data Availability

The datasets analyzed for this study can be requested from Prediction and Recognition Of Cognitive declinE through Spontaneous Speech (PROCESS) Signal Processing Grand Challenge (Tao et al., 2025) https://processchallenge.github.io/dataset/. Further inquiries can be directed to the corresponding author.

## Footnotes

1 HuBERT model: https://huggingface.co/facebook/hubert-large-ls960-ft

2 Whisper library from OpenAI: https://github.com/openai/whisper

3 See the official 2025 IEEE ICASSP PROCESS Signal Processing Grand Challenge results for the team NeuralAvengers_KozminskiUniversity at: https://processchallenge.github.io/finalresults/

## References

[B1] Boschi V. Catricala E. Consonni M. Chesi C. Moro A. Cappa S. F. (2017). Connected speech in neurodegenerative language disorders: a review. Front. Psychol. 8:269. doi: 10.3389/fpsyg.2017.00269 28321196 PMC5337522

[B2] Bucks R. S. Singh S. Cuerden J. M. Wilcock G. K. (2000). Analysis of spontaneous, conversational speech in dementia of Alzheimer type: evaluation of an objective technique for analysing lexical performance. Aphasiology 14, 71–91. doi: 10.1080/026870300401603

[B3] Buntinx F. Kester A. Bergers J. Knottnerus J. A. (1996). Is depression in elderly people followed by dementia? A retrospective cohort study based in general practice. Age Ageing 25, 231–233. doi: 10.1093/ageing/25.3.231 8670559

[B4] Chen J. Wang Y. Wang D. (2014). A feature study for classification-based speech separation at low signal-to-noise ratios. IEEE/ACM Trans. Audio, Speech, Lang. Proc. 22, 1993–2002. doi: 10.1109/TASLP.2014.2359159

[B5] Chen J. Ye J. Tang F. Zhou J. (2021). “Automatic detection of Alzheimer's disease using spontaneous speech only,” in Interspeech, 3830. doi: 10.21437/Interspeech.2021-2002 35493062 PMC9056005

[B6] Chen T. Guestrin C. (2016). “Xgboost: a scalable tree boosting system,” in Proceedings of the 22nd ACM SIGKDD International Conference on Knowledge Discovery and Data Mining, 785–794. doi: 10.1145/2939672.2939785

[B7] Cheplygina V. de Bruijne M. Pluim J. P. (2019). Not-so-supervised: a survey of semi-supervised, multi-instance, and transfer learning in medical image analysis. Med. Image Anal. 54, 280–296. doi: 10.1016/j.media.2019.03.009 30959445

[B8] Chlasta K. Wołk K. (2021). Towards computer-based automated screening of dementia through spontaneous speech. Front. Psychol. 11:623237. doi: 10.3389/fpsyg.2020.623237 33643116 PMC7907518

[B9] Cockrell J. R. Folstein M. F. (2002). “Mini-mental state examination,” in Principles and Practice of Geriatric Psychiatry, 140–141. doi: 10.1002/0470846410.ch27(ii)

[B10] Cummins N. Pan Y. Ren Z. Fritsch J. Nallanthighal V. S. Christensen H. . (2020). “A comparison of acoustic and linguistics methodologies for Alzheimer's dementia recognition,” in Proceedings of the Interspeech, 2182–2186. doi: 10.21437/Interspeech.2020-2635

[B11] Dataiku (2025). Automated machine learning — dataiku dss 14 documentation. https://doc.dataiku.com/dss/latest/machine-learning/auto-ml.html (Accessed March 7, 2025).

[B12] de la Fuente Garcia S. Ritchie C. Luz S. (2020). Artificial intelligence, speech, and language processing approaches to monitoring Alzheimer's disease: a systematic review. J. Alzheimer's Dis. 78, 1547–1574. doi: 10.3233/JAD-200888 33185605 PMC7836050

[B13] Eyben F. Scherer K. R. Schuller B. W. Sundberg J. André E. Busso C. . (2015). The geneva minimalistic acoustic parameter set (gemaps) for voice research and affective computing. IEEE Trans. Affect. Comput. 7, 190–202. doi: 10.1109/TAFFC.2015.2457417

[B14] Eyben F. Weninger F. Gross F. Schuller B. (2013). “Recent developments in opensmile, the munich open-source multimedia feature extractor,” in Proceedings of the 21st ACM International Conference on Multimedia, 835–838. doi: 10.1145/2502081.2502224

[B15] Eyben F. Wöllmer M. Schuller B. (2010). “Opensmile: the munich versatile and fast open-source audio feature extractor,” in Proceedings of the 18th ACM international conference on Multimedia, 1459–1462. doi: 10.1145/1873951.1874246

[B16] Fasnacht J. S. (2023). Conversion between the montreal cognitive assessment and the mini-mental state examination scores in older adults. J. Am. Geriatr. Soc. 71, 869–879. doi: 10.1111/jgs.18124 36346002

[B17] Ferrari A. J. Santomauro D. F. Aali A. Abate Y. H. Abbafati C. Abbastabar H. . (2024). Global incidence, prevalence, years lived with disability (YLDS), disability-adjusted life-years (dalys), and healthy life expectancy (hale) for 371 diseases and injuries in 204 countries and territories and 811 subnational locations, 1990–2021: a systematic analysis for the global burden of disease study 2021. Lancet 403, 2133–2161. doi: 10.1016/S0140-6736(24)00757-8 38642570 PMC11122111

[B18] Fowler N. R. Partrick K. A. Taylor J. Hornbecker M. Kelleher K. Willis D. R. (2025). Implementing early detection of cognitive impairment in primary care to improve care for older adults. J. Intern. Med. 298, 31–45. doi: 10.1111/joim.20098 40410933 PMC12159721

[B19] Georges J. Miller O. Bintener C. (2020). Estimating the prevalence of dementia in europe. Technical report, Alzheimer Europe.

[B20] Grand Challenge at ICASSP 2025 (2025). Process Challenge: Prediction and Recognition of Cognitive Decline Through Spontaneous Speech. Available online at: https://processchallenge.github.io/ (Accessed May 27, 2025).

[B21] Hsieh S. Schubert S. Hoon C. Mioshi E. Hodges J. R. (2013). Validation of the addenbrooke's cognitive examination iii in frontotemporal dementia and Alzheimer's disease. Dement. Geriatr. Cogn. Disord. 36, 242–250. doi: 10.1159/000351671 23949210

[B22] Hsu W.-N. Bolte B. Tsai Y.-H. H. Lakhotia K. Salakhutdinov R. Mohamed A. (2021). Hubert: self-supervised speech representation learning by masked prediction of hidden units. IEEE/ACM Trans. Audio, Speech Lang. Proc. 29, 3451–3460. doi: 10.1109/TASLP.2021.3122291

[B23] Hurst A. Lerer A. Goucher A. P. Perelman A. Ramesh A. Clark A. . (2024). GPT-4o system card. arXiv preprint arXiv:2410.21276.

[B24] Jadoul Y. Thompson B. De Boer B. (2018). Introducing parselmouth: a python interface to praat. J. Phon. 71, 1–15. doi: 10.1016/j.wocn.2018.07.001

[B25] Jarrold W. Peintner B. Wilkins D. Vergryi D. Richey C. Gorno-Tempini M. L. . (2014). “Aided diagnosis of dementia type through computer-based analysis of spontaneous speech,” in Proceedings of the Workshop on Computational Linguistics and Clinical Psychology: From Linguistic Signal to Clinical Reality, 27–37. doi: 10.3115/v1/W14-3204

[B26] Jost B. C. Grossberg G. T. (1995). The natural history of Alzheimer's disease: a brain bank study. J. Am. Geriatr. Soc. 43, 1248–1255. doi: 10.1111/j.1532-5415.1995.tb07401.x 7594159

[B27] Kashyap A. M. Rao D. Boland M. R. Shen L. Callison-Burch C. (2025). Predicting explainable dementia types with LLM-aided feature engineering. Bioinformatics 41:btaf156. doi: 10.1093/bioinformatics/btaf156 40199828 PMC12021793

[B28] Ke G. Meng Q. Finley T. Wang T. Chen W. Ma W. . (2017). “Lightgbm: a highly efficient gradient boosting decision tree,” in Advances in Neural Information Processing Systems, 30.

[B29] König A. Satt A. Sorin A. Hoory R. Toledo-Ronen O. David R. (2015). Automatic speech analysis for the assessment of patients with predementia and Alzheimer's disease. Alzheimer's Dement. 1, 112–124. doi: 10.1016/j.dadm.2014.11.012 27239498 PMC4876915

[B30] Kornblith S. Shlens J. Le Q. V. (2019). “Do better imagenet models transfer better?” in The IEEE Conference on Computer Vision and Pattern Recognition (CVPR) (IEEE), 2661–2671. doi: 10.1109/CVPR.2019.00277

[B31] Krizhevsky A. Sutskever I. Hinton G. E. (2012). “Imagenet classification with deep convolutional neural networks,” in Advances in Neural Information Processing Systems, eds. F. Pereira, C. J. C. Burges, L. Bottou, and K. Q. Weinberger (Curran Associates, Inc.), 1097–1105.

[B32] Luz S. (2017). “Longitudinal monitoring and detection of Alzheimer's type dementia from spontaneous speech data,” in 2017 IEEE 30th International Symposium on Computer-Based Medical Systems (CBMS) (IEEE), 45–46. doi: 10.1109/CBMS.2017.41

[B33] Luz S. Haider F. de la Fuente Garcia S. Fromm D. MacWhinney B. (2021b). Alzheimer's dementia recognition through spontaneous speech. Front. Comput. Sci. 3:780169. doi: 10.3389/fcomp.2021.780169 35291512 PMC8920352

[B34] Luz S. Haider F. De la Fuente S. Fromm D. MacWhinney B. (2021a). Detecting cognitive decline using speech only: the adresso challenge. arXiv preprint arXiv:2104.09356.

[B35] Luz S. Haider F. Fromm D. Lazarou I. Kompatsiaris I. MacWhinney B. (2024). An overview of the adress-m signal processing grand challenge on multilingual Alzheimer's dementia recognition through spontaneous speech. IEEE Open J. Signal Proc. 5, 738–749. doi: 10.1109/OJSP.2024.3378595 38957540 PMC11218814

[B36] Martínez-Nicolás I. Llorente T. E. Martínez-Sánchez F. Meilán J. J. G. (2021). Ten years of research on automatic voice and speech analysis of people with Alzheimer's disease and mild cognitive impairment: a systematic review. Front. Psychol. 12:620251. doi: 10.3389/fpsyg.2021.620251 33833713 PMC8021952

[B37] Mueller K. D. Koscik R. L. Hermann B. P. Johnson S. C. Turkstra L. S. (2018). Declines in connected language are associated with very early mild cognitive impairment: results from the wisconsin registry for Alzheimer's prevention. Front. Aging Neurosci. 9:437. doi: 10.3389/fnagi.2017.00437 29375365 PMC5767238

[B38] Mueller K. D. Van Hulle C. A. Koscik R. L. Jonaitis E. Peters C. C. Betthauser T. J. . (2021). Amyloid beta associations with connected speech in cognitively unimpaired adults. Alzheimer's Dement. 13:e12203. doi: 10.1002/dad2.12203 34095435 PMC8158164

[B39] Nasreddine Z. S. Phillips N. A. Bédirian V. Charbonneau S. Whitehead V. Collin I. . (2005). The montreal cognitive assessment, moca: a brief screening tool for mild cognitive impairment. J. Am. Geriatr. Soc. 53, 695–699. doi: 10.1111/j.1532-5415.2005.53221.x 15817019

[B40] Nichols E. Steinmetz J. D. Vollset S. E. Fukutaki K. Chalek J. Abd-Allah F. . (2022). Estimation of the global prevalence of dementia in 2019 and forecasted prevalence in 2050: an analysis for the global burden of disease study 2019. Lancet Public Health 7, e105–e125. doi: 10.1002/alz.051496 34998485 PMC8810394

[B41] Niu H. Álvarez-Álvarez I. Guillén-Grima F. Aguinaga-Ontoso I. (2017). Prevalence and incidence of Alzheimer's disease in Europe: a meta-analysis. Neurología 32, 523–532. doi: 10.1016/j.nrleng.2016.02.009 27130306

[B42] Paszke A. Gross S. Massa F. Lerer A. Bradbury J. Chanan G. . (2019). “Pytorch: an imperative style, high-performance deep learning library,” in Advances in Neural Information Processing Systems, 32.

[B43] Pedregosa F. Varoquaux G. Gramfort A. Michel V. Thirion B. Grisel O. . (2011). Scikit-learn: machine learning in python. J. Mach. Learn. Res. 12, 2825–2830.

[B44] Radford A. Kim J. W. Xu T. Brockman G. McLeavey C. Sutskever I. (2022). “Robust speech recognition via large-scale weak supervision,” in International Conference on Machine Learning (PMLR), 28492–28518.

[B45] Rohanian M. Hough J. Purver M. (2020). “Multi-modal fusion with gating using audio, lexical and disfluency features for Alzheimer's dementia recognition from spontaneous speech,” in Proceedings of the Interspeech, 2187–2191. doi: 10.21437/Interspeech.2020-2721

[B46] Ruzi R. Pan Y. Ng M. L. Su R. Wang L. Dang J. . (2025). A speech-based mobile screening tool for mild cognitive impairment: technical performance and user engagement evaluation. Bioengineering 12:108. doi: 10.3390/bioengineering12020108 40001628 PMC11851810

[B47] Schuller B. Steidl S. Batliner A. (2013). “The interspeech 2013 computational paralinguistics challenge: social signals, conflict, emotion, autism,” in Proceedings of INTERSPEECH, 148–152. doi: 10.21437/Interspeech.2013-56

[B48] Tao F. Mirheidari B. Pahar M. Young S. Xiao Y. Elghazaly H. . (2025). “Early dementia detection using multiple spontaneous speech prompts: the process challenge,” in ICASSP 2025–2025 IEEE International Conference on Acoustics, Speech and Signal Processing (ICASSP) (IEEE), 1–2. doi: 10.1109/ICASSP49660.2025.10889017

[B49] Vilalta-Franch J. Llinas-Regla J. López-Pousa S. (1996). The mini cognitive examination for screening in epidemiologic studies of dementia. Neurologia 11, 166–169. 8754631

[B50] Weiner J. Angrick M. Umesh S. Schultz T. (2018). “Investigating the effect of audio duration on dementia detection using acoustic features,” in Interspeech, 2324–2328. doi: 10.21437/Interspeech.2018-57

[B51] Weiner J. Engelbart M. Schultz T. (2017). “Manual and automatic transcriptions in dementia detection from speech,” in INTERSPEECH, 3117–3121. doi: 10.21437/Interspeech.2017-112

[B52] Weiner J. Herff C. Schultz T. (2016). “Speech-based detection of Alzheimer's disease in conversational German,” in INTERSPEECH, 1938–1942. doi: 10.21437/Interspeech.2016-100

[B53] Weninger F. Eyben F. Schuller B. W. Mortillaro M. Scherer K. R. (2013). On the acoustics of emotion in audio: what speech, music, and sound have in common. Front. Psychol. 4:292. doi: 10.3389/fpsyg.2013.00292 23750144 PMC3664314

[B54] Wolf T. Debut L. Sanh V. Chaumond J. Delangue C. Moi A. . (2020). “Transformers: state-of-the-art natural language processing,” in Proceedings of the 2020 Conference on Empirical Methods in Natural Language Processing: System Demonstrations (Association for Computational Linguistics), 38–45. doi: 10.18653/v1/2020.emnlp-demos.6

[B55] Wu X. Saraf P. P. Lee G. Latif E. Liu N. Zhai X. (2025). Unveiling scoring processes: dissecting the differences between llms and human graders in automatic scoring. Technol. Knowl. Learn. 1–16.

[B56] Yosinski J. Clune J. Bengio Y. Lipson H. (2014). “How transferable are features in deep neural networks?” in Advances in Neural Information Processing Systems, 3320–3328.

[B57] Yuan J. Bian Y. Cai X. Huang J. Ye Z. Church K. (2020). “Disfluencies and fine-tuning pre-trained language models for detection of Alzheimer's disease”, in Proceedings of the Interspeech, 2162–2166. doi: 10.21437/Interspeech.2020-2516

[B58] Zafar M. A. Zhang X. Shahin M. Ahmed B. (2025). “Multi-class dementia detection using acoustic features-icassp-2025 process challenge,? in ICASSP 2025–2025 IEEE International Conference on Acoustics, Speech and Signal Processing (ICASSP) (IEEE), 1–2. doi: 10.1109/ICASSP49660.2025.10889847

